# Efficacy of inhaled antibiotics in children with ventilator-associated pneumonia: A systematic review and meta-analysis

**DOI:** 10.2478/jccm-2026-0003

**Published:** 2026-01-30

**Authors:** Sher W. Chee, Rafdzah A. Zaki, Shih Y. Hng, Kah P. Eg, Qiao Y. Lee, Jessie A. de Bruyne, Anna Marie Nathan

**Affiliations:** Department of Pediatrics, Faculty of Medicine, Universiti Malaya, Kuala Lumpur, Malaysia; Department of Social and Preventive Medicine, Centre of Epidemiology and Evidence-Based Practice, Faculty of Medicine, Universiti Malaya, Kuala Lumpur, Malaysia

**Keywords:** lung infection, nebulize, aerosol, antibiotics, systematic review, pneumonia

## Abstract

**Introduction:**

The nebulization of antibiotics allows the delivery of high concentration of medication to the lungs without the systemic side-effects.

**Aims:**

We performed a systematic review and meta-analysis to determine the efficacy and safety of inhaled antibiotics in children with ventilator-associated pneumonia (VAP).

**Data sources:**

We searched Web of Science, SCOPUS, MEDLINE Complete, CINAHL and ClinicalTrials.gov trials registry until June 2025. This study was registered (CRD42024504982).

**Study selection:**

We included studies published in the last ten years that recruited children under 18 years old with VAP and treated with inhaled antibiotics. We excluded studies of children with tracheostomy and bronchiectasis.

**Data extraction:**

Type of intervention (inhaled ± intravenous (IV) antibiotics), clinical improvement, duration of mechanical ventilation (MV) and hospitalization, bacterial eradication, and adverse events were recorded.

**Results:**

Seven articles (346 patients) reported the use of inhaled antibiotics in VAP, of which four were randomized controlled trials. These studies included premature infants, neonates and children. The commonest inhaled antibiotic used was colistin (six studies). Meta-analysis revealed that inhaled antibiotics + IV antibiotics versus IV antibiotics +/− inhaled normal saline(placebo) resulted in no significant reduction in duration of MV (MD 0.88 days, 95% CI −2.72, 4.49; p=0.63, I^2^ = 85%) and ICU stay (MD 0.34[−2.79,3.40]; p=0.83, I^2^ = 80%). Clinical success (RR 0.68, 95% CI 0.39, 1.21; p=0.19, I^2^ =24%), microbiological eradication (RR 1.93, 95%CI 0.97,3.78; p=0.06, I^2^ = 2%) and mortality (RR 0.91, 95% CI 0.67, 1.24; p=0.54, I^2^ =0%) were also not significantly different. Inhaled antibiotics were not associated with increased nephrotoxicity (RR 0.91, 95% CI 0.18, 4.61; p=0.91, I^2^ = 30%).

**Conclusion:**

More robust studies are required to confirm the clinical efficacy of inhaled antibiotics in the treatment of VAP. Nonetheless, adjunctive inhaled antibiotics may be safe in children, although close monitoring is still required.

## Introduction

The treatment of ventilator associated pneumonia (VAP) caused by organisms like *Pseudomonas aeruginosa*, and *Acinetobacter spp* can be challenging and is associated with increased morbidity, mortality and healthcare costs [1, 2]. With the rise of antibiotic-resistant organisms, often treatment with high-dose, broad-spectrum antibiotics are required [3,4,5,6]. However, commonly used antibiotics like B-lactams, colistin and aminoglycosides when administered intravenously, have reduced lung bioavailability as shown by reduced concentrations of lung epithelial to plasma concentrations [7,8,9]. Furthermore, prolonged use of systemic antimicrobial therapy like aminoglycosides and polymyxins are associated with significant side-effects like nephrotoxicity and ototoxicity in both adults and children [7, 10,11,12,13].

Delivery of antibiotics through inhalation or nebulization has significant advantages over systemic antibiotic therapy in treating lower respiratory tract infections. Nebulized antibiotics can achieve a high concentration in the lung tissue, effectively reducing the bacterial load of these pathogens while reducing the risk of systemic toxic effects [8, 9, 14, 15]. These drugs, when nebulized, have shown to have negligible to low trough plasma levels [14].

A recent meta-analysis in adults, investigating the use of inhaled colistin, in the treatment of VAP, found increased microbiological clearance but no significant difference in clinical outcomes [16]. Yet in a recent multicenter study in adults, nebulized amikacin reduced the risk of developing ventilator-associated pneumonia as compared with placebo [17]. While the Infectious Disease Society of America [18] supports the use of inhaled antibiotics in VAP, the position paper by the European Society of Clinical Microbiology does not support the use of inhaled antibiotics in ventilated adults [19].

The use of inhaled antibiotics in the management of children with VAP has rarely been described and the practice is varied among intensive care units. Hence, we conducted this systematic review and meta-analysis to determine the effect of inhaled antibiotics on (a) eradicating bacteria, (b) improving clinical outcomes (days of intubation, days in ICU), (c) reducing mortality and (d) risk of adverse effects in children treated for VAP.

## Materials and methods

### Protocol and Registration

The report follows the framework of the Preferred Reporting Items for Systematic Reviews and Meta-analysis (PRISMA) 2020 statement [20]. The protocol was registered in the National Institute for Health and Care Research International Prospective Register of Systematic Reviews (PROSPERO) with registration number CRD42024504982. We excluded studies with bronchiectasis and tracheostomy-dependent children as a comprehensive review has been recently published [21].

### PICO definition

The clinical question under the PICO (Patient-Intervention-Comparator-Outcome) framework format was: For children with VAP, do inhaled antibiotics versus other treatment modalities improve the clinical, microbiological, and safety outcomes? The PICO questions are as follows:

Q: Does inhaled antibiotic treatment improve the clinical, microbiological and safety outcomes for children with VAP?
P: Children ≤ 18 years old with VAP
I: Inhaled antibiotics
C: Placebo or no therapy or other therapy (Intravenous and/or oral antibiotics)
O: Microbiological clearance, clinical success, clinical failure, duration of mechanical ventilation, duration of ICU stay, VAP and other-cause mortality, adverse effects

### Database Sources and Search Strategy

A comprehensive search of Web of Science, SCOPUS, MEDLINE Complete, AND CINAHL Complete online databases was done using the search terms (Inhaled OR aerosoli* OR nebuli*) AND (antibiotics OR antimicrobial) AND (pediatric OR pediatric OR child* OR neonate OR toddler OR adolescent) AND ventilator-associated pneumonia OR nosocomial infection OR hospital-acquired infection). Results were limited to articles published in English from 1^st^ January 2014 to 30^th^ June 2025. We also searched the ClinicalTrials.gov trials registry.

### Study Selection

Studies that fulfilled the following criteria were included: 1) Study design: All observational and randomized trials, 2) Participants: All pediatric patients with VAP, 3) Trials using inhaled antibiotics as intervention were compared with placebo, with no treatment or other routes of antibiotic administration. Exclusion criteria were: 1) Adults >18 years old, 2) Tracheostomy-dependent children and bronchiectasis (including cystic fibrosis) and 3) Case reports and case series.

Two authors (SW, AMN) screened all the studies identified in the literature search by screening the titles, abstracts and full texts using EndNote Version 21. Any disagreements between the two reviewers were resolved by a third author (RZ).

### Data extraction and Outcomes

One author extracted data and confirmed it with another. Using a pre-developed data extraction sheet in Microsoft Excel, the following data were extracted from included studies: the first author’s name, year of publication, country of origin, study design, and study population (inclusion/exclusion criteria).

The primary outcomes searched for were the (a) type of intervention (medication, dosage and duration), (b) clinical and microbiological outcomes, (c) duration of mechanical ventilation (MV), (d) duration of stay in ICU, (e)eradication of infection and (f) adverse effects including nephrotoxicity and bronchospasm, VAP-related mortality, all-cause mortality, and study limitations.

### Quality assessment

The risk of bias was assessed using the Strengthening the Reporting of Observational Studies in Epidemiology (STROBE) checklist for observational studies and the Cochrane risk of bias tool for randomized controlled trials [22]. The STROBE statement contains a 22-item checklist that guides the reporting of observational studies to facilitate critical assessment and interpretation of results. The checklist is divided into sections: title, abstract, introduction, methods, results, discussion, and other information (funding). It was decided to have scores of 0 if the item was not fulfilled, a score of 1 if the item was fulfilled, and a score of NA (non-applicable) if the item was not applicable for the specific publication. The total number of items that fulfilled the criteria was added and divided by the total number of items for each study, respectively, and multiplied by 100 to get the results in percentages [23]. Randomized trials were assessed using the Revised Cochrane risk-of-bias tool for randomized trials (RoB 2) and the Review Manager 5.4 risk of bias tool. The tool assessed the following: randomization process, deviations from intended interventions, missing outcome data, measurement of the outcome, selection of the reported result and overall bias [24]. Each item was graded to determine whether the studies were considered high, low, or unclear risk of bias. An appointed third reviewer (RZ) resolved any differences in the assessment process. Studies that had a score of <60% were considered as high risk and were excluded.

### Data Synthesis

The meta-analysis was performed when sufficient data for each outcome were reported with a similar study design. All statistical analyses were performed using Review Manager (RevMan) version 5.3 (Cochrane Collaboration, London, UK). Data Estimation and Conversion for Meta-analysis (DECoMA) version 1.0 was used for data conversion. Continuous outcomes were presented as mean and standard deviation (SD). Dichotomous data were presented as risk ratios (RR) and 95% confidence interval (CI). Meta-analysis was performed using a fixed-effect model if there was no heterogeneity between studies or a random-effect model if there was significant heterogeneity. Statistical heterogeneity was measured through the I^2^ statistic and classified as low (I^2^ < 25%), moderate (I^2^ 25–50%), or high (I^2^ > 50%).

## Results

### Search Results and Study Selection

**Fig. 1.**
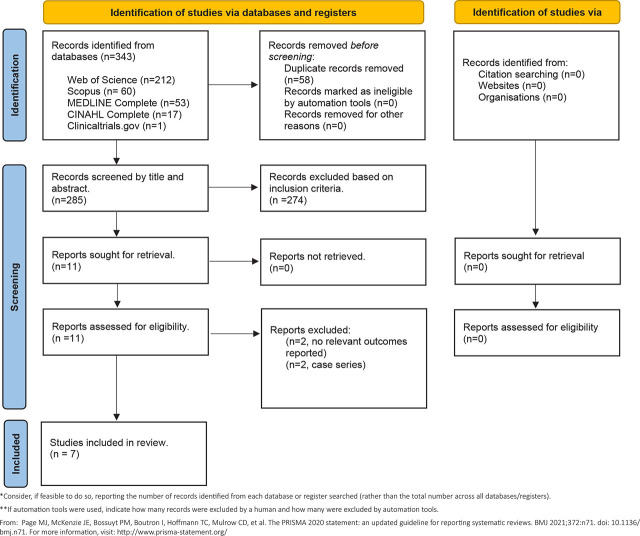
Preferred Items for Systematic Review and Meta-Analysis (PRISMA) screening process.

Our initial search of the literature yielded a total of 343 searches: Web of Science (212), Scopus (60), MEDLINE Complete (53) and CINAHL Complete (17). We also gathered data from ClinicalTrials.gov for unpublished trials and found one study of interest which has not started recruitment yet [25]. This multicenter randomized controlled trial study will evaluate the benefit of a 3-to-7-day prophylactic course of inhaled colistin vs placebo among children with VAP. [25]. After screening through duplicated records, titles and abstracts, we retrieved 11 potentially relevant full-text articles for evaluation. Four articles were excluded for reasons stated in **Figure 1**. Finally, seven studies[26,27,28,29,30,31,32] were selected for inclusion in this review.

### Study Characteristics

The definitions of clinical, microbiological outcomes and adverse events are shown in Table 1. The main characteristics and outcomes of the seven included trials are shown in Table 2 [26,27,28,29,30,31, 33]. One study was a retrospective cohort study [27], while two were retrospective case control studies [28, 33] and the remaining four were randomized controlled trials [26, 29,30,31]. Inhaled colistin was used in all six studies [27,28,29,30,31, 33]. while one study used inhaled amikacin [26]. The studies were from Ukraine, Iran, Turkey, Pakistan, India and Taiwan. None were sponsored by pharmaceutical companies. Four randomized controlled trials were included in meta-analysis [26, 29,30,31].

**Table 1.** Definition of clinical, bacteriological and safety outcomes in the SEVEN included studies

**Study/year/country**	**Clinical outcome**	**Microbiological outcome**	**Adverse events**
Levchenko 2023/Ukraine	Clinical parameters (body temperature and changes in respiratory parameters while on ventilator, including C dyn, PEEP and PIP), laboratory indices (blood oxygen saturation, leukocyte count), instrumental methods (CXR) Average duration of MV Average duration of ICU stays	Microbial load in sputum from endotracheal tubes on Day 3 and Day 5	NA
Polat 2015/Turkey	Favourable clinical response: Clinical cure or clinical improvement (complete or partial resolution, respectively, of presenting symptoms and signs of pneumonia w/o requirement of any additional antibiotics at the end of the colistin treatment Clinical failure: persistence or progression of presenting symptoms and signs of pneumonia without requirement of any additional antibiotics at the end of colistin treatment Duration of PICU stay and Duration of MV after the initiation of colistin VAP-related mortality: patients who died due to persisting or worsening signs of pneumonia while receiving colistin tx Other-cause mortality: patients who died due to all other non-VAP related causes	Bacterial eradication – no growth of the causative microorganisms in the final culture Bacterial failure – persistence of the causative microorganism on follow-up cultures regardless of the clinical outcome Recurrence – re isolation of the same pathogen regardless of the clinical outcome Time to bacterial eradiation (TBE): duration of colistin tx until the day of bacterial eradication, defined as the day the cultures first became negative and remained negative in repeated samples	Nephrotoxicity – 50% or greater increased in serum creatinine level from the baseline and/or elevation of serum creatinine values beyond the estimated normal rage for the patient’s age group at any time during the tx Neurotoxicity Bronchoconstriction
Hussain 2020/Pakistan	Clinical success was defined as Clinical improvement: incomplete resolution OR Clinical cure: complete resolution Clinical failure: worsening or persistence Duration of NICU stay and Duration of MV after the initiation of colistin VAP-related mortality was defined as neonatal death during colistin therapy with persistent signs of pneumonia Overall Mortality	Eradication of the causative organism – no growth of the pathogen in the final culture of specimens during the entire hospitalization Persistence of the causative organism – persistent growth of the causative pathogen regardless of the clinical outcome Regrowth of the causative organism – re-isolation of the same pathogen regardless of the clinical outcome of the infection Colonization – persistence or regrowth of the pathogen without symptoms and signs of infection	Acute kidney injury (AKI) – increment in serum creatinine levels of ≥0.3mg/dl within 48 hours or 150–200% increase from baseline trough value Neurotoxicity – an episode of seizure or change in consciousness level at any time during colistin therapy Electrolyte imbalance causing additional electrolyte replacement required: Hypomagnesemia: <1.6mg/dl, Hypokalaemia: <3.5mM/L
Khanababee 2024/Iran	Days of hospital stay Days of MV Fever occurrence Laboratory markers: White blood cell (WBC) counts, Erythrocyte sedimentation rate (ESR), C-reactive protein (CRP) levels Mortality rate	No microbiological outcomes measured	Nephrotoxicity Neurotoxicity Tachycardia Bronchospasm
Bharathi 2022/India	Favorable outcome: Presenting symptoms and signs of infection were completely or partially resolved at the termination of colistin treatment OR Normalization of WBC counts, improvement of ABG, and reduction or disappearance of radiological findings on CXR Average duration of MV Average postoperative ICU stays Average hospital stays (days) VAP associated mortality Other cause mortality	Bacterial eradication – final culture of specimens demonstrating no growth of the pathogen during the entire hospitalization Persistence – Persistent growth of the responsible pathogen regardless of the clinical outcome of the infection Recurrence (regrowth) – reappearance of the same pathogen regardless of the clinical outcome Colonization – persistence or reappearance of the same pathogen with no symptoms and signs of infection	Nephrotoxicity – Change in serum creatinine levels
Sachev 2019/India	Clinical cure was defined as complete resolution of all signs and symptoms of pneumonia, with normalization of total leucocyte count and temperature at the end of CS therapy. Clinical failure was defined as persistent or worsening of presenting signs and symptoms. Duration on MV and PICU stay. Mortality rate	Bacterial eradication, if no growth of the same pathogen as in the last culture of a respiratory specimen; Persistent bacteria if same microbe was obtained along with signs and symptoms of VAP infection;	Renal impairment was defined if increase in the serum creatinine level of patients occurred with previously normal renal function or a doubling of the baseline serum creatinine level in patients with pre-existing renal insufficiency. Adverse events.
Kang 2014/Taiwan	NA	Cure was defined as at least three separate sputum culture that had no A.baumannii growth and each at least 1 day apart	Renal compromise – presence of decreased urine output ≤1ml/kg/hr, plasma creatinine level ≥1.5mg/dl and elevated BUN (ml/kg/hr)

### Methodological Quality Assessment

Supplementary Table S1 presents the full risk of bias assessments

Outcome measures (See Table 2)

**Table 2.** Ventilator-associated pneumonia - main characteristics, outcomes and results of the included studies

**Author/Year/Country**	**Study design**	**Patients**	**Type of Nebulizer**	**Intervention/Placebo**	**Duration & dosage**	**Results reported**
**Inhaled + IV Abx VS IV Abx only, n=4**						
Levchenko 2023/Ukraine	Randomized controlled trial, single-blind (Pilot study)	20 (10/10) infants with VAP in ICU	Not stated	TR1 (n=10): inhaled AMK + systemic IV Abx TR2 (n=10): systemic IV Abx only	TR1: Inhaled AMK 500mg BD for x1/52 + IV MER (5 pt), IV CPZ-SBT (3 pt), IV AZM (2 pt) TR2: CPZ-SBT (which was changed into IV MER 3 days later) and CLI (4 pt), AZM and CLI (3 pt), MER and VAN (3 pt)	A 2-fold increase in resistance and a significant increase in PIP values (22–23 cm H20) were found in the control group. Prolongation of the purulent-inflammatory process in the lungs compared to the patients administered inhaled amikacin in the early period of VAP. Decreased microbial load in sputum from the endotracheal tubes on the 3^rd^ day of antimicrobial therapy in TR1 vs TR2: (log (3.59±0.32) CFU/ml) vs ((log (5.49±0.27) CFU/ml) (P<0.001). ^*^Duration of MV days was median (range) [6(5–10) vs 7(6–12), P<0.05] Duration of ICU stay was 1 day lower in TR1 compared to TR2 [7(6–12) vs 8(7–14), P=0.20]
Polat 2015/Turkey	Retrospective Cohort study	50 (18/32) aged 1 month to 18 years critically ill children with VAP due to COS GNB in PICU	Vibrating Mesh Nebulizer	TR1 (n=18): inhaled + IV CS. TR2 (n=32): IV CS only	TR1: Inhaled CS administered concurrently >1yo: 75mg BD ≤1yo: 4mg/kg/dose BD + IV CS median dose 3.4mg/kg for a median duration of 14 days TR2: IV CS median dose 3.2mg/kg for a median duration of 16 days Both treatment arms have other concomitant antibiotic treatment (carbapenems, glycopeptides, aminoglycosides, CPZ-SBT, PIP-TAZ, fluoroquinolones)	No significant differences in favorable clinical response (P=0.362) and bacterial eradication (P=0.362) Median time to bacterial eradication (TBE) shorter in TR1 group vs TR2: median of 3 days vs median of 6 days (P<0.001) Duration of PICU stay and duration of MV was not significant between TR1 and TR2 (29 vs 26 days, P=0.8; 19 vs 22.5 days, P=0.156). VAP asstd mortality: TR1 17% vs TR2 18.8% (P=0.99) Other cause mortality: TR1 27.7% vs 18.8% (P =0.48) Only one pt in TR2 developed nephrotoxicity on treatment Day 8, however the pt also received concomitant vancomycin and radiocontrast agent on Day 7. Three pts in TR1 experienced bronchoconstriction and desaturation at the time of administration of the first doses, which did not require discontinuation of colistin treatment and was alleviated with B2-agonist.
Hussain 2020/Pakistan	Retrospective case control study	32 (16/16) neonate with MDR-assoc VAP in NICU	Not stated	TR1 (n=16): Inhaled + IV CS TR2 (n=16): IV CS only	TR1: Inhaled CS 4mg/kg/dose BD for median duration 7.5 days + IV CS 2.5–5.0mg/kg/day 2–4 daily doses for median duration 4.5 days TR2: IV CS 2.5–5.0mg/kg/day 2–4 daily doses for median duration 12.5 days Both groups have concurrent other IV Abx therapy (MER, AMK, CIP)	Clinical success: Clinical cure was significant in TR1 group compared to TR2 group (56.3% vs 31.3%, P<0.05) while clinical improvement was not significantly different (P>0.999). Eradication of infection was significant in TR1 group compared to TR2 group (68.8% vs 43.8%, P<0.05). Duration of NICU stay was not significant in TR1 group compared to TR2 group (19.5 vs 23.5 days, P=0.078). Duration of MV was significantly reduced in Inhaled + IV CS group compared to IV CS group (median range 7.5 vs 11.5 days, P<0.001) VAP asstd mortality: 12.5% in TR1 vs 31.3% in TR2 (P=0.08) Overall Mortality: 25% in TR1 vs 73.8% in TR2 (P=0.06) AKI occurrence in inhaled + IV CS group compared to IV CS group only was 1 and 5 neonates respectively (6.25% vs 31.3%, P<0.05)
Khanababee 2024/Iran	Randomized controlled trial study	80 (40/40) children aged 2–18 yrs old with MDR-assoc VAP in PICU (excluded patients who died [n =2])	Not stated	TR1 (n=40): Inhaled CS + IV Abx TR2 (n=40): IV Abx only	TR1: Inhaled CS 3 to 5mg/kg every 6 hours for a duration of 2 to 3 weeks (maintained at least 2 weeks) + IV Abx TR2: IV Abx only	Fever occurrence was significantly higher in the control group than in the colistin group (7.5% vs. 2.5%, P = 0.04). No significant in white blood cell (WBC) counts between TR1 and TR2 (13,120 ± 7,821.08 and 21,871.79 ± 35,818.61 mm^3^, P = 0.31). No significant differences between the two groups in erythrocyte sedimentation rate (ESR) and C-reactive protein (CRP) levels (P > 0.05). Duration of hospital stay was not significant between TRI and TR2 (29.83 ± 22.35 vs. 34.92 ± 20.03 days, P = 0.29). Days of mechanical ventilation were not significantly different (23.15 ± 21.17 vs. 24.06 ± 17.87 days, P = 0.83). No significant difference in mortality rate (26 children [65%] in TR2 and 23 children [57.5%] in TR1 died [P=0.49]). No neurotoxicity was observed in either group. Nephrotoxicity was reported at 2.5% in TR1 and 7.5% in TR2, though not statistically significant (P = 0.305). Tachycardia was not significant when TR1 compared to TR2 (80% vs 70%, P=0.302)
**Inhaled + IV Abx vs Inhaled NS + IV Abx, n=2**						
Bharathi 2022/India	Randomized double-blinded controlled study	98 (51/47) children with VAP due to GNB in the postoperative period following cardiac surgery for congenital heart disease in SICU	Breath-actuated jet nebulizer	TR 1 (n=51): Inhaled CS + IV Abx TR2 (n=47): Inhaled NS + IV Abx	TR 1: Inhaled CS 4mg/kg BD reconstituted in 4ml of sterile NS + IV Abx TR 2: Inhaled sterile NS 4ml BD + IV Abx IV Abx used in both groups are CS, antipseudomonal Abx such as MER, AMK, CPZ-SBT, PIP-TAZ, LZD and TIG. Received both IV colistin + one antipseudomonal antibiotic: TR1 47.1% vs TR2 44.6% Received IV colistin only: TR1 17.65% vs TR2 38.2% Received IV antipseudomonal only: TR1 35.25% vs TR2 17.2% *Duration of Inhaled CS and NS was administered until the end of systemic antibiotic therapy.*	Favorable clinical response was not significant (mean 37 vs 31 days, P=0.696) ^**^Significant reduction in duration of MV with (mean 11.2 vs 18.1 days, P=0.002), postoperative ICU stay (mean 14.04 vs 22.3 days, p=0.004) and hospital stay (mean 17.6 vs 26.2 days, P=0.005) in TR1 compared to TR2. Eradication of GNB causing VAP in 80.4% of the patients in TR1 and 68.1% patients in TR2 (P=0.16) VAP-related mortality: TR1 3.9% vs TR2 14.9% (P=0.07) Other cause mortality: TR1 23.5% vs TR2 19.1% (P=0.64) ^**^ No statistically significant differences in adverse events between the two groups (but no details regarding this).
Sachdev 2019/India	Open-label, pilot randomized controlled trial	35 (16/19) children with MDR-asstd VAP in PICU	Vibrating mesh nebulizer (Aerogen, Ireland)	TR1 (n-16): Inhaled CS + IV antibiotics TR2 (n=19): Inhaled NS + IV antibiotics	TR1: Inhaled CS 500,000 IU reconstituted in 4ml of NS (TDS) + IV antibiotics TR2: Inhaled NS 4ml TDS + IV antibiotics *Duration of Inhaled CS and NS was administered until the end of systemic antibiotic therapy. IV antibiotics were given for at least 14 days.*	Clinical cure was higher in TR1 compared to TR,2 however was not significant (93.7% vs 73.6%, p=0.12) No significant difference in bacterial eradication (100% vs 76.5%, p=0.06) Microbiological cure was assessed in 13 and 17 in TR1 and TR2 respectively due to early extubation/BBS culture negative/isolates resistant to colistin ^*^Median (IQR) of MV days: TR1 15(11.5,17) vs TR2 11(6.5,20.5), p=0.128 Median (IQR) PICU days: TR1 16(14,22) vs TR2 13(11,36), p=0.185 Mortality rate was higher in TR2 as compared to TR1 (31.5% VS 18.7%, P=0.39) although not statistically significant. No statistically significant differences in adverse events (cough, bronchospasm, renal impairment) between the two groups.
**Inhaled Abx only vs IV Abx only, n-1**						
Kang 2014/Taiwan	Retrospective case control	31 (8/23) preterm infants with VAP due to *A.baumannii* infection in NICU	Neb-easy nebulizer kit	TR1 (n=8°): Inhaled CS TR2 (n=23): IV Abx only °4 pts received concomitant IV Abx but did not improve until inhaled antibiotics was started	TR1: Inhaled CS 33.4mg BD with average 9.1 days (range 4-22 days) with 4 pts received IV antimicrobial concurrently TR2: Combination of antibiotics regimen: IV AMK (4 pt), AMP-SBT (14 pt), IV CAZ (6 pt), IV MER (7 pt), IV CEF (2 pt)	All pre-term infants in both treatment groups were reported to be cured with *A.baumannii* eradicated from airway secretions and discharged. No adverse effects reported

TR1: Treatment regimen 1 (main); TR2: Treatment regimen 2 (control); VAP: Ventilator-associated pneumonia; COS GNB: Colistin-organism sensitive gram negative bacteria; MDR: Multi-drug resistant; SICU: Surgical Intensive Care Unit; NICU: Neonatal intensive care unit; OD: Once daily; BD: Twice daily; Abx: Antibiotic; IT Abx: Intrathecal Antibiotic; 1yo: 1 year old; ; MV: Mechanical ventilation; Pt: patient; Tx: treatment; Pa: *pseudomonas aeruginosa*; *H.influenza: Haemophilus influenza; A.baumannii: Acinetobactor baumannii*; BAL: Bronchoalveolar lavage; PO: Per oral; BBS: Blind bronchial sampling.

AMK: Amikacin; AZM: Aztreonam; CPZ: Cefoperazone; AMP: Ampicillin; SBT: Sulbactam; CAZ: Ceftazidime; CEF: Cefepime; CLI: Clindamycin; CS: Colistin; MER: Meropenam; VAN: Vancomycin; CIP: Ciprofloxacin; PIP-TAZ: Piperacillin-Tazobactam; LZD: Linezolid; TIG: Tigecyclin

* *Details obtained from personal communication with the authors.*

** *Standard deviation not stated*

### Ventilator-associated pneumonia

#### a. Study group (Inhaled + IV Abx) vs Control group (IV Abx only)

Four studies looked at the combination of inhaled and intravenous antibiotics versus intravenous antibiotics only [26,27,28,29]. Two were retrospective [27, 28] while two were a randomized controlled study [26, 29]. Results are summarized in Table 2.

Two retrospective studies investigated the use of inhaled colistin in children with ventilator-associated pneumonia (VAP) in the NICU and ICU [27, 28]. Two studies included children under one-year-old, and inhaled colistin was administered at 4mg/kg BD [27, 28]. In the study that included children over one-year-old too, inhaled colistin was administered at 75mg BD [27]. Another study only included children aged 2–18 years old and administered inhaled colistin 3 to 5mg/kg every 6 hours for a duration of two to three weeks [29].

Polat et al. showed a significant reduction in time to bacterial eradication (TBE) in the study group compared to the control group (3 vs 6 median days, p<0.001) [27]. However, there were no significant differences in clinical response, bacterial eradication and mortality (VAP related mortality and other-cause mortality). Conversely, Hussain et al. showed that the use of inhaled and IV antibiotics resulted in significantly more cases with clinical cure (9 vs 5 cases, p<0.05) and eradication of infection (68.8% vs 43.8%, p<0.05) [28]. However, clinical improvement was similar (4 vs 4 cases, p>0.999). As for mortality, there was a trend towards reduced mortality in those with adjunct inhaled antibiotics: VAP (12.5% vs 31.3%, p =0.08), overall mortality (25% vs 73.8%, p=0.06).

A randomized controlled study recently published in 2024 investigated the use of inhaled colistin in ICU [29]. Khanababee et al. showed fever occurrence was higher in control group compared to study group (7.5% vs 2.5%, P =0.04). However, other outcomes measured (MV and hospital stay days, mortality, infective blood parameters, fever duration, were not significant). The types of co-administered intravenous antibiotics which were not described in the study. Microbiological outcomes were also not measured.

Worth mentioning here is another randomized, controlled, single-blind (pilot) study which compared the administration of adjunctive inhaled antibiotics (amikacin 500mg BD) for one week combined with IV antibiotics versus IV antibiotics only, in mechanically ventilated infants with VAP [26]. This study investigated the respiratory parameters while on a ventilator, MV and ICU stay duration, and microbial load in the sputum pre-and post-treatment. A 2-fold increase in resistant organisms and a significant increase in peak inspiratory pressure (PIP) values was found in the control group. There was a significant reduction in early apoptotic and necrosis of circulating leukocytes in the study group compared to the control group. The average (range) duration of MV was also significantly reduced in the study group compared to the control group 6 (5–10) vs 7 (6–12) days, p<0.05, while the duration of stay in the ICU was not significant 7 (6–12) vs 8 (7–14) days, p=0.20 (personal communication in author) [26]. Levchenko et al. also found a significant reduction in microbial load in the endotracheal tube cultures of the study group on the third day of antibiotic therapy (log (3.59±0.32) CFU/ml) compared to the control group (log (5.49±0.27) CFU/ml) (p<0.001) and the results were reproduced on the fifth day as well. This study did not report on bacterial eradication nor adverse events [26].

#### b. Study group (Inhaled Abx + IV Abx) vs Control group (Inhaled Normal Saline + IV Abx)

There were two studies that used Inhaled NS in the control group [30]. A prospective, randomized, double-blind, controlled study compared the concomitant use of inhaled colistin (4mg/kg BD) with IV antibiotics (n=51) and Inhaled NS with IV antibiotics (n=47) in children with VAP in the postoperative period following cardiac surgery in a surgical ICU. The study group had a significant decrease in the duration of MV (11.2 vs 18.1 days, p=0.002), postoperative ICU stay (14.04 vs 22.3 days, p=0.004), and total hospital stay (17.6 vs 26.2 days, p=0.005) compared to the control group. However, there was no significant difference in favorable clinical response (37 vs 31, p=0.696), bacteriological outcome (80.4% vs 68.1%, p=0.16). There was a trend to reduced risk of VAP-related mortality (3.9% vs 14.7%, p=0.07) but not significant other-cause mortality (p=0.64). Adverse effects of inhaled colistin were not seen in both groups despite not using prophylactic B2 agonists. Regarding nephrotoxicity, differences in serum creatinine levels were insignificant between the two groups (mean 0.18 vs 0.12, p=0.081). In another study by Sachdev et al., who performed a randomized controlled trial in 35 children with inhaled colistin 500,000IU given 8hrly together with IV antibiotics, found no significant difference in outcomes (bacterial eradication, duration of MV and ICU stay, mortality, clinical cure).

#### c. Study group (Inhaled Abx only) vs Control group (IV Abx only)

Only one study used inhaled antibiotics only in the intervention group [33]. This retrospective case-control looked at the role of inhaled colistin monotherapy for VAP of *Acinetobacter baumannii* in premature infants. They recruited 31 preterm infants in which eight patients were given inhaled colistin 33.4mg BD (equivalent to 1 million IU), and 23 patients received IV antibiotics only. Four of the eight patients who received inhaled colistin initially received IV antibiotics but did not improve; hence, inhaled colistin was started. All preterm infants in both treatment groups were reported to be microbiologically cured with *Acinetobacter baumannii* eradicated (at least three negative sputum cultures and each at least one day apart) from airway secretions and discharged. This study found that inhaled colistin was safe to use in prematurity as no clinical or renal function abnormalities were noted. None of the patients had adverse events from the inhaled colistin therapy despite not using bronchodilators.

### Adverse effects

Hussain et al. showed acute kidney injury (AKI) was more prevalent in the control group compared to the study group (31.3% vs 6.25%, p<0.05); this could be due to the longer duration of IV colistin given in the control group compared to study group (12.5 vs 7.5 days) [28]. Polat et al. showed that one child developed nephrotoxicity in the control group [27]. However, this patient also received concomitant vancomycin and radiocontrast agents before IV colistin. Bharathi et al found no significant differences in serum creatinine levels between the two groups (mean 0.18 vs 0.12, p=0.081)[30]. Khanababee et al. found no significant difference in nephrotoxicity or serum creatinine levels between two groups despite the high dose (3–5 mg/kg inhaled every 6 hrs) and prolonged use (2–3 weeks) of inhaled colistin [29]. Sachdev et al found no significance difference in renal impairment [31]. The study in premature infants also found no clinical or renal function abnormalities [33].

Bronchoconstriction was an adverse effect of inhaled colistin therapy, as observed in Polat et al., where three patients experienced bronchoconstriction and desaturation after receiving the first dose. However, this problem was alleviated with B2-agonist and well tolerated after that [27]. In contrast, Hussain et al, Bharati et al and Khanababee et al did not report any bronchoconstriction, although it was not evident in the study whether all patients were routinely prescribed B2-agonists before inhaled antibiotics [28,29,30]. Sachdev et al pre-treated patients with hyper-reactive airways with inhaled salbutamol. They found no increased risk of bronchospasm in the treatment group [31]. The study by Levchenko did not comment on side-effects [26]. In premature infants, no bronchospasm was noted despite not using bronchodilators [33].

Polat et al. and Khanababee et al. did not report any cases of neurotoxicity. In contrast, Hussain et al. reported three cases when comparing both treatment arms (one in the study group versus two in the control group, p<0.782).

Finally, in the study that used inhaled colistin in premature infants, no clinical or renal function abnormalities were noted. None of the patients had adverse events from the inhaled colistin therapy despite not using bronchodilators [33].

### Quantitative (meta-analysis) and descriptive analysis for VAP in children

We included four randomized controlled studies [27,28,29, 31] in the meta-analysis. However, not all of the outcomes could be included into the meta-analysis due to differential reporting or lack of reporting standard deviation or range. Levchenko et al did not report on clinical success, bacterial eradication, nor mortality, while Khanababee et al did not report on clinical success or bacterial eradication. Bharathi et al, did not provide standard deviation for analysis of MV, ICU stay, and lacked details regarding adverse events. We finally decided to combine the control arms of IV antibiotics with and without inhaled NS when analyzing MV, ICU stay, mortality, and nephrotoxicity, as inhaled NS was considered a placebo.

**Fig. 2.**
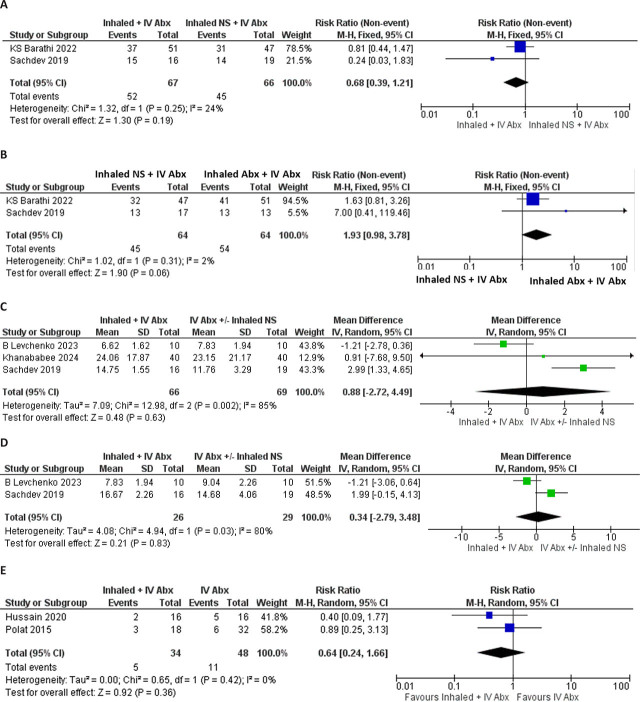
Forest plot for clinical outcomes. (A) Clinical success, (B) Bacterial Eradication (C) Duration of Mechanical Ventilation (D) Duration of intensive care unit (ICU) stay (E) Mortality

We examined the following clinical outcomes: clinical success (cure or improvement), eradication of bacteria, duration of MV, nephrotoxicity, and other-cause mortality, as shown in Figure 2A–E.

Analyses of studies from Bharati et al. and Sachev et al. found no significant improvement in clinical success (RR 0.68, 95% CI 0.39–1.21; p = 0.19; I^2^ = 24%) in the groups with inhaled + IV antibiotics (Figure 2A).

Similarly, studies by Bharati et al. and Sachev et al. found that the likelihood of eradicating bacteria in the combined inhaled + IV antibiotics group (RR 1.93, 95%CI 0.97,3.78; p=0.06, I^2^ = 2%) was possibly higher than with IV antibiotics +Inhaled NS alone; however, the difference did not achieve statistical significance. **Figure 2B**

However, the combination of inhaled + intravenous antibiotics compared to intravenous antibiotics +/− inhaled NS had no significant reduction in the duration of MV (MD 0.88 days, 95% CI −2.72, 4.49; p=0.63, I^2^ = 85%) and ICU stay (MD 0.34[−2.79,3.40]; p=0.83, I^2^ = 80%) (Figure 2C and 2D respectively).

As for VAP-related mortality (RR 0.64, 95% CI 0.24, 1.66; p=0.36, I^2^=0%) and other-cause mortality (RR 0.91, 95% CI 0.36, 2.33; p=0.85, I^2^=40%), both outcomes were not significantly different between the two treatment groups **(Figure 2E).**

### Adverse events

**Fig. 3.**
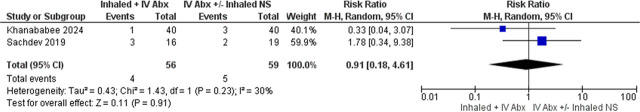
Forest plot for nephrotoxicity

Inhaled + IV antibiotics therapy was associated with a non-significant reduced risk of nephrotoxicity (RR 0.20, 95% CI 0.08, 1.14; p=0.08, I^2^=0%) (Figure 3).

## Discussion

This systematic review aimed to determine the efficacy of inhaled antibiotics in children with VAP, associated with gram-negative bacteria (GNB) in children. We found seven studies involving neonates and children and the commonest antibiotic used was inhaled colistin, which was included in six trials [27,28,29,30,31, 33], while one trial [26] used inhaled amikacin. Four studies used Inhaled and IV antibiotics versus IV antibiotics alone while the other two used IV antibiotics with inhaled normal saline (NS) as a placebo. The meta-analysis included four randomized controlled studies, however due to difference in reporting outcomes, majority of the analysis was on two studies. We found no significant improvement in clinical success and no reduction in bacterial eradication, mechanical ventilation, ICU stay nor mortality. Importantly, there was no significant increase in adverse effects.

### Efficacy of Inhaled Antibiotics in VAP

#### Mechanical ventilation and intensive care unit stay

In the meta-analyses, adjunctive inhaled antibiotics did not significantly shorten the duration of MV nor ICU stay. Others have shown a reduction in MV and ICU stay, by about 4 days each, albeit both of these studies were retrospective [27, 28]. Bharathi et al. also demonstrated significant reduction in duration of MV by 7 days, postoperative ICU days by 8 days and total hospitalization by 9 days, in the group receiving inhaled and intravenous antibiotics compared to the control group [30]. However, we could not include Bharathi’s study into the meta-analyses as standard deviations were not reported in the paper. Considering that Bharathi et al had 98 patients in their study, exclusion of this may have affected our finding. Also to note was high heterogeneity in both these analyses. This may have resulted in our negative outcome. Reducing duration of invasive ventilation and ICU stay has both clinical and financial implications [34]. There may be other factors that influence duration of ICU stay like central line infections, renal failure, tracheostomy, need for external ventricular shunts and surgical procedures [35, 36]. Only half of the studies compared the underlying illness of patients, which ultimately may be a confounding factor associated with the duration of ICU stay and/or mechanical ventilation [27, 28, 31, 32]. In adults, two systematic reviews by Qin et al (13 studies involving 1733 patients, looking at adjunctive use of Amikacin) and Zampieri et al (12 studies involving 885 patients, looking at both adjunctive and substitutive use of amikacin, colistin and tobramycin) showed no significant effect on duration of MV and ICU stay, although there was high heterogeneity in both these outcomes [11, 37]. Adults have significant co-morbidities like diabetes and poor cardiac function that may affect both the duration of MV and ICU stay too [38, 39].

#### Clinical success, bacterial eradication & mortality

Our meta-analyses involving studies from Bharati et al [30] and Sachdev et al [31] found no significant increase in clinical success. Clinical success was similarly defined in the study by Polat et al and Hussain et al [27, 28], of whom only Hussain et al found a significant improvement in clinical cure. Hussain differed from the others as he studied neonates in the NICU, while the others studies patients post cardiac surgery or in the PICU with various illnesses. This is probably the reason for the difference in findings. As mentioned earlier, underlying disease may potentially influence clinical outcomes. In adults, the reviews by Qin and Zhang found differing results in clinical responses: RR 1.23 (95% CI 1.13–1.34) and OR 1.39 (95% CI 0.87–2.20) [11, 16].

As for bacterial eradication, the meta-analyses showed a trend to increased microbiological clearance (RR 1.93, p=0.06), although it did not achieve statistical significance. In the retrospective studies not included in this meta-analysis, both Polat el al and Hussain et al found bacterial eradication was increased when inhaled colistin was used [27, 28]. Similarly, Levchenko et al. found a significant decrease of microbial load in sputum from the endotracheal tubes on the third and fifth day in the inhaled amikacin + IV antibiotics group compared to IV antibiotics only group [26]. The lack of significance in bacterial clearance across most studies could be due to the variation in duration of therapy, as most of the studies continued inhaled antibiotics as long as IV antibiotics were on board [27, 30]. However, Hussain et al., who continued inhaled antibiotic treatment alone for at least 3 days after the cessation of IV antibiotics, found significant bacterial eradication [28]. Results on bacterial eradication could also be affected by when the culture was taken or analyzed e.g. early in the course of treatment versus later after treatment, whereby recrudescence of bacteria is more likely to occur. Interestingly, inhaled colistin monotherapy was used for VAP of *Acinetobacter baumannii* in premature infants, and all preterm infants reported to be microbiologically cured (at least three negative sputum cultures and each at least one day apart) [33]. In children, there are no head-to-head studies between colistin and amikacin. In adults, the review by Sella et al (11 studies involving 1472 patients, looking at the adjunctive use various inhaled antibiotics (amikacin, polymyxin B, colistin and tobramycin), found increased bacterial eradication, with OR 2.63(95% CI 1.36–5.09) [40]. They also found that the effect size on bacterial clearance was higher with colistin compared to amikacin (OR 2.21 [95%CI 1.25–3.92] vs RR 1.51[95%CI 1.35–1.69]) respectively [40].

As for VAP-related mortality and other-cause mortality, we decided to combine the results of studies which included inhaled NS as this is a placebo, and there was low heterogeneity. Three studies were analyzed and there were no significant differences between the treatment and control groups. This is probably due to the small numbers of children with mortality in these studies. In adults, effect sizes for pneumonia-cause of mortality by Qin et al (for amikacin) and Valachis et al (both adjunctive and substitutive use of colistin) were RR 1.12 (95% CI 0.82–1.52) and OR 0.58 (95% CI 0.34–0.96) respectively [11, 41]. As for all-cause mortality, the effect size for amikacin by Qin et al and colistin by Vardakas et al (13 studies involving 1135 patients, adjunctive use of colistin) were not significant at RR 1.17 (0.98–1.50) and RR 0.94 (0.81–1.08) respectively [11, 42].

Our negative meta-analysis suggests that studies should focus on the use of inhaled antibiotics on specific populations, such as premature infants alone, solid organ transplant recipients, or post-cardiac surgery, who have an increased risk for prolonged IMV, hence at risk of developing MDR strains, which may result in more positive findings. Furthermore, the use of inhaled antibiotics for prevention rather than for treatment may be a more effective strategy [25, 43].

### Doses of inhaled antibiotics

Expert opinion recommends that inhaled colistin + systemic colistin may be considered for VAP caused by XDR GNB especially when the pathogen is only sensitive to colistin and if there is treatment failure with systemic colistin alone [44]. We recommend that the dose for adjunctive inhaled colistin in infants and children is 4mg/kg BD, as observed in the two studies in infants [27, 28] and one study in children [30]. In preterm infants with VAP due to *A. baumannii*, inhaled colistin was administered at 1.0 M IU BD (33.33mg) [33]. This is also in line with the recommendation of the Children’s Antimicrobial Management Program (ChAMP) Monograph by Perth Children Hospital [45].

The duration of inhaled colistin observed among the four studies on VAP varied greatly [27, 28, 30, 33]. We found that cessation of combination treatment following resolution of clinical signs and symptoms did not achieve significant bacterial clearance based on three studies[27, 30, 31]. , while continuing inhaled antibiotics alone after combination therapy for a minimum of three days resulted in bacterial clearance, as shown in Hussain et al. [28]. Hence, we recommend that duration of inhaled antibiotics should be continued longer than systemic antibiotics to achieve bacterial clearance.

### Adverse Events

Bronchospasm has been reported to occur in patients with and without asthma [46]. However, our review showed that this can be prevented by using a B2-agonist, without the need to discontinue the drug [27]. This review also did not find consistent reporting of bronchospasm as an adverse event. Even in children with CF, adverse events from the use of inhaled antibiotics are rarely reported [47]. This shows that bronchospasm from the use of inhaled antibiotics is not common in young children. This is different in adults, whereby bronchospasm seems to be more common with risks of RR 2.55 (95%CI 1.40–4.66) and OR 5.19 (95% CI 1.05–25.52) [11, 16]. Use of preservative free antibiotics do have a lower risk for bronchospasm [48].

The common adverse reactions described with intravenous colistin treatment are nephrotoxicity and neurotoxicity due to its narrow therapeutic index [49]. As for nephrotoxicity, this meta-analysis and the other studies [27, 28, 30, 33], which were not included in the quantitative analysis, observed that this risk was not significantly different between the study and control groups. Even in premature infants, the use of colistin has been reported to be effective [50, 51] and safe [51,52,53]. Given that colistin is negatively charged at human physiological pH, the negative charge of the alveolar basement membrane likely contributes to the slow systemic passage of colistin into the systemic circulation [54]. Colistin-associated nephrotoxicity can still occur in up to 10% of the cases, as shown in a review involving 104 critically ill children with normal renal function and colistin dosage administered at 5mg/kg per day in three divided doses [55]. Conversely, studies in adults have found reduced nephrotoxicity with the use of inhaled antibiotics[56]. Nephrotoxicity and neurotoxicity associated with intravenous colistin are related to the amount of daily maintenance dose and not to the loading or cumulative dose and are usually reversible [57]. Higher or excessive doses of colistin (>5mg/kg ideal body weight/day) are associated with an increased risk of nephrotoxicity and often result from the use of actual body weight in obese patients [58]. Nonetheless, careful surveillance and the use of preventative strategies for antibiotic related toxicity (such as nephrotoxicity and ototoxicity) are still essential to avoid possible toxicity [59].

With the use of nebulized antibiotics, antibiotic resistance is a significant concern. This was not reported in any of our included studies. In a small study involving children with tracheostomy, one out of six developed microbial resistance, after a median duration of 74 days of inhaled antibiotics. [60]. In adults with VAP, the systematic review showed reduced emergence of resistant bacteria strains (RR 0.18, 95% CI 0.05–0.64) [15]. A systematic review (19 studies) on adults with stable NCFB revealed a higher risk of isolating resistant organisms with inhaled antibiotic treatment [61]. The pooled risk ratio was 1.86 (95% CI, 1.51–2.30; p<0.001, I^2^:6%), and resistance increased regardless of which inhaled antibiotic was used (p=0.20, I^2^: 35%) [61]. However, a review that explored the use of inhaled tobramycin, colistin and gentamicin in adults with NCFB did not demonstrate any significant emergence of antimicrobial isolates in sputum, and any increase in minimum inhibitory concentrations (MIC) was transient with a return to baseline after discontinuation of treatment [55]. Although antibiotic resistance is a concern, evidence shows that inhaled antibiotics could eradicate existing multidrug-resistant organisms (MDRO) in intubated patients and reduce the pressure for the selection of new resistant organisms whilst reducing the use of systemic antibiotics [62].

### Drug delivery in ventilated patients

One should be wary of other essential aspects of nebulized therapy e.g. type of device used and administration techniques, to improve drug deposition balanced with safety and patient comfort [8, 63]. An ultrasonic mesh nebulizer, placed in the inspiratory limb 15-40 cm before the Y-piece and removing the heat and moisture exchange during therapy, is important. Use of a smooth inner surface tubing will also improve drug deposition [8]. Placing a filter in the expiratory limb, between the ventilator during nebulization, which should be changed after treatment, will prevent expiratory limb obstruction.

### Limitations

The limited number of studies with small sample sizes and few randomized controlled trials in children with VAP will impact the generalizability and reliability of the findings. Furthermore, the use of inhaled antibiotics in various diseases and populations would have impacted the outcome. Our meta-analysis on the four RCTs still only included a maximum of three studies, with some analyses showing significant heterogeneity between these studies.

## Conclusion and recommendations

Using inhaled antibiotics with systemic antibiotics in children with ventilator-associated pneumonia did not result in statistically significant outcomes. We observed a minor, non-significant decrease in days on bacterial eradication, but no notable changes in clinical outcomes like MV, ICU stay, clinical success, or mortality. There was no increase in side effects, such as bronchospasm or nephrotoxicity, however close monitoring of each individual patient is still required.

Larger randomized studies on specific populations are needed to confirm that inhaled antibiotics improve outcomes for children with VAP. Studies directly comparing amikacin and colistin are also important to determine if colistin is the best choice.
